# Theory and Simulation of Nanostructures

**DOI:** 10.3390/nano11092202

**Published:** 2021-08-27

**Authors:** Sotirios Baskoutas

**Affiliations:** Department of Materials Science, University of Patras, 26504 Patra, Greece; bask@upatras.gr

The intense interest in nanostructured materials is fueled by tremendous economic and technological benefits anticipated from nanotechnology and nanodevices. Nanostructured materials have demonstrated great potential for applications in optoelectronics, sensors, engineering and cancer therapy. The advance in these areas will affect our daily lives from how we design a fast computer and a material with specific properties to how we preserve the environment, and how we diagnose and treat diseases and pollution.

The present section ‘Theory and Simulation of Nanostructures’ has the aim to publish papers that promote the field of computational materials science through the application of modern and state-of-the-art computational methods alone or in combination with experimental techniques to discover and design new nanomaterials with tailored properties suitable for applications mainly in optoelectronics, sensors, engineering and cancer therapy. The computational methods include density functional theory (DFT), time-dependent density functional theory (TTDFT), empirical pseudopotential methods, Monte Carlo simulations, finite element methods and effective mass approximation. 

Some of the problems which are still under investigation concern the design and development of semiconductor nanostructured materials with a controllable band gap which are more efficient as materials for entangled photon pair generation and materials for single-photon sources with huge applications in quantum information science. Quantum information science will offer potential to substantially change the face of modern optoelectronics and make new optoelectronic devices (e.g., lighting devices based on light-emitting diodes, infrared lasers in an optical fiber network, lasers for recording/retrieving information on/from optical storage media, solar photovoltaic cells and many others) more efficient, targeted and successful, and thus more affordable. 

From the other side, manufacturing of reliable sensors consisting of inexpensive nanomaterials receives great interest in today’s technology. Hence, the current demand for sensor technology requires ultra-low cost materials with exceptional sensing and mechanical or thermal stability. 

Furthermore, the design of new engineering nanomaterials is one of the greatest achievements in our daily lives, and it has been focused on the evolution, prosperity, security and quality of life of humans since the beginning of history. These new nanomaterials with tailored properties pave the way to the development of new technologies, whether they are in civil, chemical, construction, nuclear, aeronautical, agricultural, mechanical or electrical engineering.

Finally, recent advances in nanomedicine raise exciting possibilities for future nanoparticle applications in personalized cancer therapy, and new strategies for building hybrid nanostructures are offering interesting platforms such as effective multimodality cancer imaging (i.e., photothermal, photoacoustic and magnetic resonance imaging) and combinational cancer therapy (i.e., photothermal ablation of tumors, photodynamic therapy and targeted delivery-based chemotherapy).

The research interest of the section ‘Theory and Simulation of Nanostructures’ includes, but is not limited to, the following:

- Nanomaterials for quantum technology applications;
- Nanomaterials for sensor applications;
- Nanomaterials for engineering applications;
- Nanomaterials for health science.

